# Antifungal susceptibility profiles and drug resistance mechanisms of clinical *Candida duobushaemulonii* isolates from China

**DOI:** 10.3389/fmicb.2022.1001845

**Published:** 2022-12-05

**Authors:** Xin-Fei Chen, Han Zhang, Xin-Miao Jia, Jin Cao, Li Li, Xin-Lan Hu, Ning Li, Yu-Ling Xiao, Fei Xia, Li-Yan Ye, Qing-Feng Hu, Xiao-Li Wu, Li-Ping Ning, Po-Ren Hsueh, Xin Fan, Shu-Ying Yu, Jing-Jing Huang, Xiu-Li Xie, Wen-Hang Yang, Ying-Xing Li, Ge Zhang, Jing-Jia Zhang, Si-Meng Duan, Wei Kang, Tong Wang, Jin Li, Meng Xiao, Xin Hou, Ying-Chun Xu

**Affiliations:** ^1^Department of Laboratory Medicine, State Key Laboratory of Complex Severe and Rare Diseases, Peking Union Medical College Hospital, Chinese Academy of Medical Science and Peking Union Medical College, Beijing, China; ^2^Graduate School, Chinese Academy of Medical Science and Peking Union Medical College, Beijing, China; ^3^Beijing Key Laboratory for Mechanisms Research and Precision Diagnosis of Invasive Fungal Diseases, Beijing, China; ^4^Medical Research Center, Peking Union Medical College Hospital, Chinese Academy of Medical Science and Peking Union Medical College, Beijing, China; ^5^Jinling Hospital Institute of Clinical Laboratory Science, School of Medicine, Nanjing University, Nanjing, Jiangsu, China; ^6^Department of Dermatology, Hua Shan Hospital, Fudan University, Shanghai, China; ^7^Department of Laboratory Medicine, Fujian Provincial Hospital, Fuzhou, China; ^8^Department of Laboratory Medicine, West China Hospital, Sichuan University, Chengdu, China; ^9^Department of Laboratory Medicine, Ruian People's Hospital, Wenzhou, China; ^10^Department of Laboratory Medicine, The First Medicine Center, Chinese PLA General Hospital, Beijing, China; ^11^Department of Laboratory Medicine, Zhejiang Provincial People's Hospital, Hangzhou, China; ^12^Department of Laboratory Medicine, The People’s Hospital of Liaoning Province, Shenyang, China; ^13^Department of Laboratory Medicine, No.908 Hospital of Joint Logistics Support Force, Nanchang, China; ^14^Department of Laboratory Medicine and Internal Medicine, National Taiwan University Hospital, National Taiwan University College of Medicine, Taipei, Taiwan; ^15^Department of Laboratory Medicine and Internal Medicine, China Medical University Hospital, China Medical University, Taichung, Taiwan; ^16^Department of Infectious Diseases and Clinical Microbiology, Beijing Institute of Respiratory Medicine and Beijing Chao-Yang Hospital, Capital Medical University, Beijing, China; ^17^Department of Laboratory Medicine, Peking University Third Hospital, Beijing, China

**Keywords:** *Candida duobushaemulonii*, antifungal susceptibility, *FUR1*, whole genome sequence, drug resistance mechanisms

## Abstract

*Candida duobushaemulonii*, type II *Candida haemulonii* complex, is closely related to *Candida auris* and capable of causing invasive and non-invasive infections in humans. Eleven strains of *C*. *duobushaemulonii* were collected from China Hospital Invasive Fungal Surveillance Net (CHIF-NET) and identified using matrix-assisted laser desorption/ionization time-of-flight mass spectrometry (MALDI-TOF), VITEK 2 Yeast Identification Card (YST), and internal transcribed spacer (ITS) sequencing. Whole genome sequencing of *C*. *duobushaemulonii* was done to determine their genotypes. Furthermore, *C*. *duobushaemulonii* strains were tested by Sensititre YeastOne™ and Clinical and Laboratory Institute (CLSI) broth microdilution panel for antifungal susceptibility. Three *C*. *duobushaemulonii* could not be identified by VITEK 2. All 11 isolates had high minimum inhibitory concentrations (MICs) to amphotericin B more than 2 μg/ml. One isolate showed a high MIC value of ≥64 μg/ml to 5-flucytosine. All isolates were wild type (WT) for triazoles and echinocandins. *FUR1* variation may result in *C*. *duobushaemulonii* with high MIC to 5-flucytosine. *Candida duobushaemulonii* mainly infects patients with weakened immunity, and the amphotericin B resistance of these isolates might represent a challenge to clinical treatment.

## Introduction

*Candida duobushaemulonii* belongs to the *Candida haemulonii* species complex, along with *Candida haemulonii* and *Candida haemulonii* var. *vulnera*. Yeasts belonging to this complex are closely related to the notorious *Candida auris*, which has attracted global attention with multi-drug resistant and widely disseminating (Du et al., 2020). *Candida duobushaemulonii* was initially classified as type II of *Candida haemulonii* complex. It was clearly identified as *C*. *duobushaemulonii* in 2012 (Cendejas-Bueno et al., 2012). The conventional panels used in routine microbiology laboratories often misidentify these species, making it hard to identify accurately (Fang et al., 2016; Ambaraghassi et al., 2019; Frias-De-Leon et al., 2019). Therefore, their actual incidence and global prevalence may be underestimated.

A retrospective study found that *C*. *duobushaemulonii* was first isolated in foot ulcers in 1996, where it was recovered from the toenail of a patient from Bizkaia, Spain (Jurado-Martin et al., 2020). The first isolate in China was collected under the China Hospital Invasive Fungal Surveillance Net (CHIF-NET) project in 2010 (Hou et al., 2016a). However, as an emerging species, it has been reported that fluconazole, amphotericin B, and echinocandins non-wild-type (non-WT) *C*. *duobushaemulonii* have been identified (Cendejas-Bueno et al., 2012), and the mechanism of *C*. *duobushaemulonii* with high MIC for antifungal drugs is still unclear.

Although currently reported cases of *C*. *duobushaemulonii* in China are few, hospital outbreaks of *C*. *duobushaemulonii* have been reported (Gade et al., 2020). Therefore, we conducted antifungal drug susceptibility testing and whole-genome sequencing of *C*. *duobushaemulonii* in China for 8 years. The aims were to confirm whether *C*. *duobushaemulonii* had broken out in China, and to discover the underlying mechanism of its resistance to antifungal drugs.

## Materials and methods

### Ethics statement

This study was approved by the Human Research Ethics Committee of Peking Union Medical College Hospital (No. S-263). Written informed consent was obtained from all the patients who participated in this study, aimed at culturing and studying the isolates obtained from them for scientific research.

### Fungal isolates

During the period from 2010 to 2017 (Table 1), 11 *C*. *duobushaemulonii* isolates were collected from nine different hospitals in eight provinces under the CHIF-NET. These isolates were mainly of invasive fungal infection specimens. Strains isolated before 2015 were identified and their susceptibility tested in our previous article (Hou et al., 2016a).

**Table 1:** List of isolates included in the study.

Strain	Age/Gender	Year	Source of the isolate	Clinical diagnosis	Vitek 2 (Score)	Mating type
F4468	59/male	2010	Blood	Abdominal cavity infection	*Candida duobushaemulonii* (93%)	α
F4458	36/female	2012	Blood	Breast cancer	Low Discrimination	α
F4464	56/male	2012	Blood	Common bile duct	*Candida duobushaemulonii* (95%)	α
F4490	78/male	2014	Venous catheter	Lung infection	*Candida duobushaemulonii* (88%)	α
F4566	57/female	2015	Blood	moderately severe Acute pancreatitis	*Candida duobushaemulonii* (96%)	α
F4572	45/male	2015	Ascitic fluid	HBV-related liver cirrhosis	*Candida duobushaemulonii* (97%)	α
F4586	36/female	2016	Puncture fluid	Acute myeloid leukemia	*Candida haemulonii* (87%)	α
F4608	48/male	2016	BALF^a^	Lung infection	Unidentified	α
F4560	70/male	2016	Tissue	pyogenic Osteomyelitis	*Candida duobushaemulonii* (88%)	α
F4616	51/female	2017	Tissue	Granulomatous angiitis	*Candida duobushaemulonii* (95%)	α
F7396	57/male	2017	Catheter	Cerebral hemorrhage	*Candida duobushaemulonii* (97%)	α

a BALF, bronchoalveolar lavage fluid.

### Species identification

All *C*. *duobushaemulonii* were identified at the species level using Autof-MS 1000 (Autobio, Zhengzhou, China) and Vitek MS (bioMérieux, Marcy l’Étoile, France), and confirmed by sequencing the rDNA internal transcribed spacer region (ABI 3730XL, Thermo Fisher Scientific, Cleveland, OH, United States). PCR and sequencing of the amplicons were performed using the former primers (Zhang et al., 2014; Hou et al., 2016b). All 11 isolates were also re-identified using the Vitek 2 YST Card by VITEK 2 (9.02 version, bioMérieux, Marcy l’Etoile, France) following the manufacturer’s instructions.

### DNA extraction and whole-genome sequencing

The whole genomic DNA of *C*. *duobushaemulonii* was extracted by the sodium dodecyl sulfate (SDS) method (Lim et al., 2016). The DNA library was constructed using NEBNext® Ultra™, following the manufacturer’s instructions. Agilent 2100 Bioanalyzer was used for quality confirmation. Whole genome of *C*. *duobushaemulonii* was sequenced using Illumina NovaSeq 6000 at Beijing Novogene Bioinformatics Technology Co., Ltd. Illumina reads from this study were deposited at National Center for Biotechnology Information (NCBI) under BioProject PRJNA883504. In addition, we downloaded the genome data of *C*. *duobushaemulonii* from the NCBI SRA database as described by Gade et al. (2020).

### Genome variation, phylogenetic, and population genetic analyses

Paired-end sequences with greater than 100X coverage were used for Bioinformatics analysis. *Candida duobushaemulonii* B09383 (GenBank accession number PKFP00000000.1) was used as the reference genome for analysis (Chow et al., 2018). We used BWA 0.5.9 and SAMtools and bcftools 0.1.19 to analyze single nucleotide polymorphism (SNP) and insertion-deletion (indel) (Li and Durbin, 2009a; Li et al., 2009b). SNP and Indel function annotation analysis were used snpeff 4.3 (Cingolani et al., 2012). Phylogenetic tree was constructed using RAxML 8.2.12 based on 1,000 bootstrap replicates by maximum likelihood method to investigate the *C*. *duobushaemulonii* genetic relationships (Stamatakis, 2014). The genome-wide nucleotide diversity (Pi) and the average Tajima’s D estimate were calculated by DNASP 6 (Rozas et al., 2017).

### Chromosome structure analysis and mating type analysis

We used YMAP to perform Copy Number Variation (CNV) analysis of *C*. *duobushaemulonii* (Abbey et al., 2014). We checked the BAM (Binary Alignment Map) file of *C*. *duobushaemulonii* genome by SAMtools to determine the coverage depth of the region where the MTLα gene is located, and determine the mating type of *C*. *duobushaemulonii*.

### Broth microdilution antifungal susceptibility testing

*Candida duobushaemulonii* strains were tested by Sensititre YeastOne (Thermo Scientific, Cleveland, OH, United States). In addition, the standard antifungal susceptibility testing was performed according to CLSI M27-A3. Essential agreement (EA) is defined as the percent of all Sensititre™ YeastOne™ MIC results within one 2-fold dilution of the CLSI MIC result. *Candida krusei* ATCC 6258 and *Candida parapsilosis* ATCC 22019 were selected for quality control. The epidemiological cutoff values (ECV) and clinical breakpoints of antifungals against *C*. *duobushaemulonii in vitro* have been established by the CLSI (CLSI, 2020). Among them, fluconazole MICs of greater than 32 μg/ml is considered as non-WT for *C*. *duobushaemulonii* and *C*. *auris*. Flucytosine MIC values (≥32 μg/ml) were interpreted according to the CLSI document M27-S3 (CLSI, 2008). In addition, MIC of ≥2 μg/ml was used for interpreting “resistance” of amphotericin B (Pfaller et al., 2012).

### Identification of variations associated with high MICs for *Candida duobushaemulonii*

We analyzed the mutations in *ERG11*, *FUR1*, and other genes of interest in the pathways related to sterol metabolism and 5-flucytosine metabolism (Supplementary Table S1; Arendrup and Patterson, 2017; Berkow and Lockhart, 2017).

### Review of *Candida duobushaemulonii* infections reported in PubMed

This literature review considered the available data regarding the susceptibility of the *C*. *duobushaemulonii* species to antifungals. The literature search was performed on June 26, 2022, using the following three databases: PubMed,^1^ Web of Science,^2^ and Embase.^3^ The terms “*Candida duobushaemulonii*” were entered in the category of “Title/Abstract” in the PubMed Advanced Search Builder, and “TS = (*Candida duobushaemulonii*)” was entered in the Web of Science databases. The search in Embase was conducted in the advanced search area, including the terms “‘*Candida duobushaemulonii*’: ab,ti..”

## Results

### Isolates information

Of all 11 cases, seven were male and four were female, with an average age of 54 years. Among the specimens, blood specimens accounted for four patients, tissue culture specimens for two patients, bronchoalveolar lavage fluid (BALF) culture specimens, ascitic fluid, catheter, venous catheter, and puncture fluid specimens for one patient, respectively (Table 1). The patients belonged to the following departments: medicine department (45.5%; 5/11), surgery department (45.5%; 5/11), and emergency intensive care unit (9.1%; 1/11).

### Species identification of *Candida duobushaemulonii* using MALDI-TOF, ITS sequencing and Vitek 2

All 11 clinical isolates were identified as *C*. *duobushaemulonii* by the Autof MS 1000 and Vitek MS. The ITS sequences of the study isolates exhibited over 99.5% identity to the corresponding ITS sequences of the reference *C*. *duobushaemulonii* CBS7798^T^ isolates. For Vitek 2 system, eight *C*. *duobushaemulonii* could be identified accurately, one could not be identified, one identified with low discrimination and the remaining one was misidentified as *C*. *haemulonii* (score = 87%; Table 1).

### Phylogenetic relationships and genetic diversity among *Candida duobushaemulonii*

**Figure 1:**
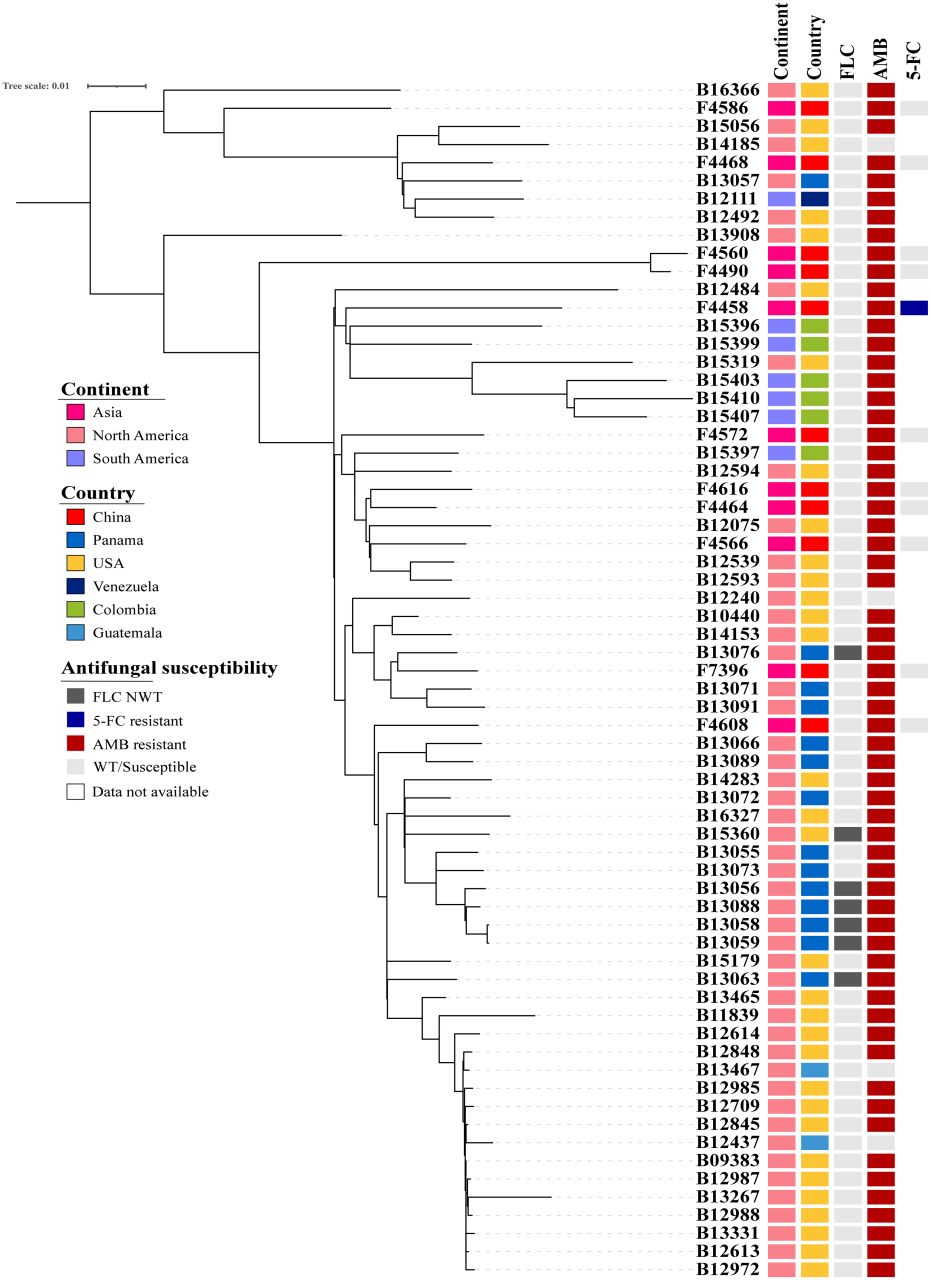
Phylogenetic tree of *Candida duobushaemulonii*. The phylogenetic tree was constructed with RAxML using the maximum likelihood method based on 8,117 SNPs. Phylogenetic tree detailing susceptibility to fluconazole (FLC), amphotericin B (AMB), and 5-flucytosine (5-FC).

*Candida duobushaemulonii* isolated in China shows no clustering distribution, and its evaluation did not exhibit clustered outbreaks (Figure 1). Based on the number of SNPs that differ between the Chinese strains and the international strains with a very little difference, it can be seen that the evolution rate of *C*. *duobushaemulonii* is very slow. The average pairwise distance between *C*. *duobushaemulonii* isolates was 700 SNPs (range: 78–1,271). The average number of nucleotides is close to the previously reported average (Gade et al., 2020). All strains in the phylogenetic tree can be divided into two clades (Figure 1). The first clade includes strains isolated from China, the United States, Guatemala, Venezuela, and Panama. The second clade includes strains isolated from China, the United States, Guatemala, Colombia, and Panama. The strains around the world presented a scattered distribution. Genome-wide diversity estimates show reduced polymorphism in *C*. *duobushaemulonii* (Pi = 0.24013), but the average Tajima’s D estimate was −0.89987 expected population expansion.

### Chromosome variation and mating type

Analysis of large fragments of *C*. *duobushaemulonii* chromosomes showed neither copy number variation nor aneuploidy in genome (Supplementary Figure S1). All *C*. *duobushaemulonii* isolates were mating type alpha.

### Antifungal susceptibility

The quality control strains (*Candida krusei* ATCC 6258 and *Candida parapsilosis* ATCC 22019) showed MICs within the expected ranges. Aggregated MIC distributions of nine antifungal agents of *C*. *duobushaemulonii* isolates by YeastOne™ are shown in Table 2. All strains were WT to fluconazole, voriconazole, itraconazole, posaconazole, caspofungin, anidulafungin, and micafungin. MIC of 5-flucytosine for one strain was >64 μg/ml, while that of the remaining strains were all less than 0.12 μg/ml. All the 11 isolates tested showed high amphotericin B MICs (MIC ≥4 μg/ml).

**Table 2:** Epidemiological cutoff values (ECV) of nine antifungal agents based on aggregated minimum inhibitory concentration distributions for *C*. *duobushaemulonii*.

	MIC (μg/ml)												MIC_50_	MIC_90_	Range	Mode^a^	ECV (μg/ml)^b^
	0.015	0.03	0.06	0.12	0.25	0.5	1	2	4	8	>8	>64					
Fluconazole						1	1	6	3				2	4	0.5–4	2	≥32
Voriconazole	3	4	2	2									0.03	0.12	0.015–0.12	0.03	≥0.5
Itraconazole		3	6	1	2								0.06	0.12	0.03–0.25	0.06	≥1
Posaconazole	8	1	2										0.015	0.06	0.015–0.06	0.015	≥1
Caspofungin	7	3	1										0.015	0.03	0.015–0.06	0.015	≥0.25
Micafungin	1	6	4										0.03	0.06	0.015–0.06	0.015	≥0.5
Anidulafungin	2	2	4	3									0.06	0.12	<0.015 to 0.12	0.06	≥1
5-flucytosine			10									1	<0.06	<0.06	<0.06 to >64	0.06	NA
Amphotericin B									3	5	3		8	>8	4 to >8	>8	NA

a Mode: Most frequent MIC.

b Calculated ECVs comprising ≥ 95% of the statistically modeled MIC population.

### Agreement between the CLSI method and sensititre YeastOne™

The EA values of the MICs between the CLSI method and YeastOne™ for most of the antifungal drugs tested were > 90%. 100% EA values were obtained for amphotericin B and 5-flucytosine. EA values for anidulafungin and micafungin were 36.4 (4/11) and 27.3% (3/11), respectively (Supplementary Table S2).

### Potential variation linked to 5-flucytosine and amphotericin B resistance

Compared with the reference genome of B09383, which is sensitive to azoles and echinocandins, we found that the amphotericin B-resistant *C*. *duobushaemulonii* isolated from China shows unique variation. We found that F4490 and F4560 have a novel mutation (V907A) in the *HMG1* gene and F4468 and F4586 has a previously reported mutation of S54N. In the *ERG20* gene, we found two novel mutations, K347N in F4468 and M101T in F4608. In the *UPC2* gene, both F4490 and F4608 possess A100T mutation. Interestingly, we discovered a novel mutation in the initiation codon (ATG-- > ATA) of *FUR1* gene in a strain (F4458) with high MIC for 5-flucytosine (Table 2). In addition, it is interesting that we found that seven strains carried A626Y, T637I or P1042A substitutions in FKS1p and V30M, A485V and/or H352R in FKS2p. However, all strains were WT to echinocandins (Supplementary Table S1).

### Literature review

Relatively limited data of 15 articles on antifungal susceptibility information for *C*. *duobushaemulonii* were reviewed. Some strains exhibited high MICs to fluconazole alone or to all azoles, and carried varation in Erg11p (Gade et al., 2020). In addition, there are three research reported emergence of echinocandin-resistant *C*. *duobushaemulonii*. To date there has been only one report on emergence of flucytosine-resistant *C*. *duobushaemulonii* strains (Supplementary Table S2).

## Discussion

*Candida duobushaemulonii,* belongs to type II *Candida haeumlonii* complex, is relative of *C*. *auris* and *Candida pseudohaemulonii*. Literature reveals that *C*. *duobushaemulonii* was wrongly identified as *C*. *haemulonii, Candida intermedia*, and *Debaryomyces hansenii* (Desnos-Ollivier et al., 2008; Fang et al., 2016; Jurado-Martin et al., 2020). Previous studies have also shown that the identification ability of MALDI-TOF needs to be improved (Hou et al., 2016a). In the present study, although ITS sequencing, Autof MS 1000, and Vitek MS system have achieved good identification results, but three *C*. *duobushaemulonii* strains could not be identified by the Vitek 2 Compact system, which database includes *C*. *auris*, *C*. *duobushaemulonii*, and *C*. *haemulonii* var. *vulnera*. Considering, MALDI-TOF and ITS sequencing techniques are not all available in routine microbiology laboratories and *C*. *duobushaemulonii* actual incidence might be underestimated.

One case of hospital transmission of *C*. *duobushaemulonii* has been reported (Gade et al., 2020). Therefore, we conducted a genetic relationship analysis of *C*. *duobushaemulonii* isolated from China. We found that there was no obvious hospital infection transmission in China. However, considering the low isolation rate of *C*. *duobushaemulonii* in China, a large data sample is needed for analysis. In the overall genome evolution, the average SNP of *C*. *duobushaemulonii* is similar to that described by Gade et al. (2020).

In the drug susceptibility test, Gade *et al* reported that only 12.7% (7/55) strain as non-WT to fluconazole, and the MICs of these strains ranged from 64 to 256 μg/ml, with six isolates from Panama and one isolate from Texas, United States (Gade et al., 2020). De Almeida *et al* found that four *C*. *duobushaemulonii* isolated in Brazil has high MICs to azole and amphotericin B (de Almeida et al., 2016). Ramos *et al* has been reported echinocandins-resistance strains isolated in Brazil (Ramos et al., 2022). Regretfully, previous studies lacked 5-flucytosine antifungal drug sensitivity and only 18.2% (2/11) *C*. *duobushaemulonii* tested were 5-flucytosine-resistance (Cendejas-Bueno et al., 2012; de Almeida et al., 2016; Ramos et al., 2022). In the literature review, we can see that despite the low isolation rate of *C*. *duobushaemulonii*, strains resistant to azoles, echinocandins, amphotericin B, or 5-flucytosine have been emerging. However, our research found that all *C*. *duobushaemulonii* are WT to all azoles. Although there were seven isolates have missense mutations in the *FKS1* and *FKS2* genes, all strains were WT to echinocandins. In addition, our study might be the first to report the high MIC of 5-flucytosine for a strain isolated from China (MIC >64 μg/ml). All strains were with high MIC range to amphotericin B (4 to >8 μg/ml), which is consist with previous reports of *C*. *duobushaemulonii* high MIC to amphotericin B (Ramos et al., 2022). Although 5-flucytosine and amphotericin B lack the interpretation breakpoint, *C*. *duobushaemulonii* is notable for the high MICs of 5-flucytosine and amphotericin B.

Compared with *C*. *auris*, *C*. *duobushaemulonii* fails to attract the attention of the whole world. *C*. *duobushaemulonii* is resistant to amphotericin B and 5-flucytosine, but the resistance mechanism is not well understood. In our study, there is no missense mutation in the common drug resistance gene *ERG11*, and aneuploidy and multiple copies were also not found in *C*. *duobushaemulonii*, which may be different from that in *C*. *auris* and *C*. *haemulonii*, with a quite different resistance mechanism as reported previously (Gade et al., 2020). *Candida duobushaemulonii* not only shows high MIC of 5-flucytosine, but also shows high MIC of amphotericin B. For 5-flucytosine, there is a missense mutation G3A (M1I) in the *FUR1* gene in the drug-resistant strains. This mutation is a completely new site and has not been reported. In addition, the resistance mechanism of amphotericin B is also worthy of attention. The mechanism of *C*. *duobushaemulonii* with high MIC to amphotericin B remains to be elusive. Although we found mutations involving sterol synthesis pathway genes in five strains, there were still six strains without mutations. In literature review, only 11.1% (11/99) *C*. *duobushaemulonii* had a lower amphotericin B MIC (<4 μg/ml). In addition, Carolus et al. founded the cell membrane sterols profile of *C*. *duobushaemulonii* was similar to amphotericin B-resistant species with mutations in *ERG2, ERG3*, *ERG6*, and *ERG11* (Silva et al., 2020). Thus, *C*. *duobushaemulonii* maybe possess high MIC to amphotericin B. Our study is similar to the previous studies in terms of shortcomings. Due to the lack of clinical treatment information, the correlation between the non-WT *C*. *duobushaemulonii* and the clinical treatment and prognosis needs further study.

In conclusion, the emergence of *C*. *duobushaemulonii*, a rare amphotericin B, and 5-flucytosine resistant fungus, is a potential threat. The phenotype of *C*. *duobushaemulonii* resistant to 5-flucytosine might be due to the variations in *FUR1*. Although the invasive infection of *C*. *duobushaemulonii* is very rare, it still needs our attention due to its drug resistance. Due to the lack of clear clinical treatment data, it is necessary to study *in vitro* the relationship between drug resistance and clinical treatment effect in the future.

## Data availability statement

The whole genome sequence raw reads presented in the study are deposited in the NCBI, BioProject PRJNA883504.

## Ethics statement

Written informed consent was not obtained from the individual(s) for the publication of any potentially identifiable images or data included in this article.

## Author contributions

X-FC, HZ, and X-MJ conceived and designed the experiment. JC, LL, X-LH, NL, Y-LX, FX, L-YY, Q-FH, X-LW, L-PN provided isolates. X-FC, HZ, X-MJ, XF, XH, SY-Y, J-JH, W-HY, X-LX, Y-XL, GZ, J-JZ, S-MD, WK, TW, and JL performed the experiments. X-FC, HZ, and X-MJ analyzed the data and wrote the manuscript. MX, Y-CX, XH and P-RH revised the manuscript. All authors contributed to the article and approved the submitted version.

## Funding

This work was supported by the National Natural Science Foundation of China (82002178), National High Level Hospital Clinical Research Funding (2022-PUMCH-B-074), CAMS Innovation Fund for Medical Sciences (2021-I2M-1-038 and 2021-I2M-1-044), and Beijing Key Clinical Specialty for Laboratory Medicine-Excellent Project (No. ZK201000).

## Conflict of interest

The authors declare that the research was conducted in the absence of any commercial or financial relationships that could be construed as a potential conflict of interest.

The reviewer XL declared a shared affiliation with the authors X-FC, HZ, X-MJ, S-YY, J-JH, X-LX, W-HY, Y-XL, GZ, J-JZ, S-MD, WK, TW, JL, MX, and Y-CX to the handling editor at the time of review.

## Publisher’s note

All claims expressed in this article are solely those of the authors and do not necessarily represent those of their affiliated organizations, or those of the publisher, the editors and the reviewers. Any product that may be evaluated in this article, or claim that may be made by its manufacturer, is not guaranteed or endorsed by the publisher.

## Footnotes

^1^ https://pubmed.ncbi.nlm.nih.gov

^2^ https://webofknowledge.com

^3^ https://www.embase.com

## Supplementary material

SUPPLEMENTARY FIGURE S1 No obvious copy number variation in *Candida duobushaemulonii*.
