# Supplementary figures and images for: Antifungal susceptibility profiles and drug resistance mechanisms of clinical Candida duobushaemulonii isolates from China
Source: Front Microbiol. 2022 Dec 5;13:1001845. doi: 10.3389/fmicb.2022.1001845 (PMC9760970; doi:10.3389/fmicb.2022.1001845)

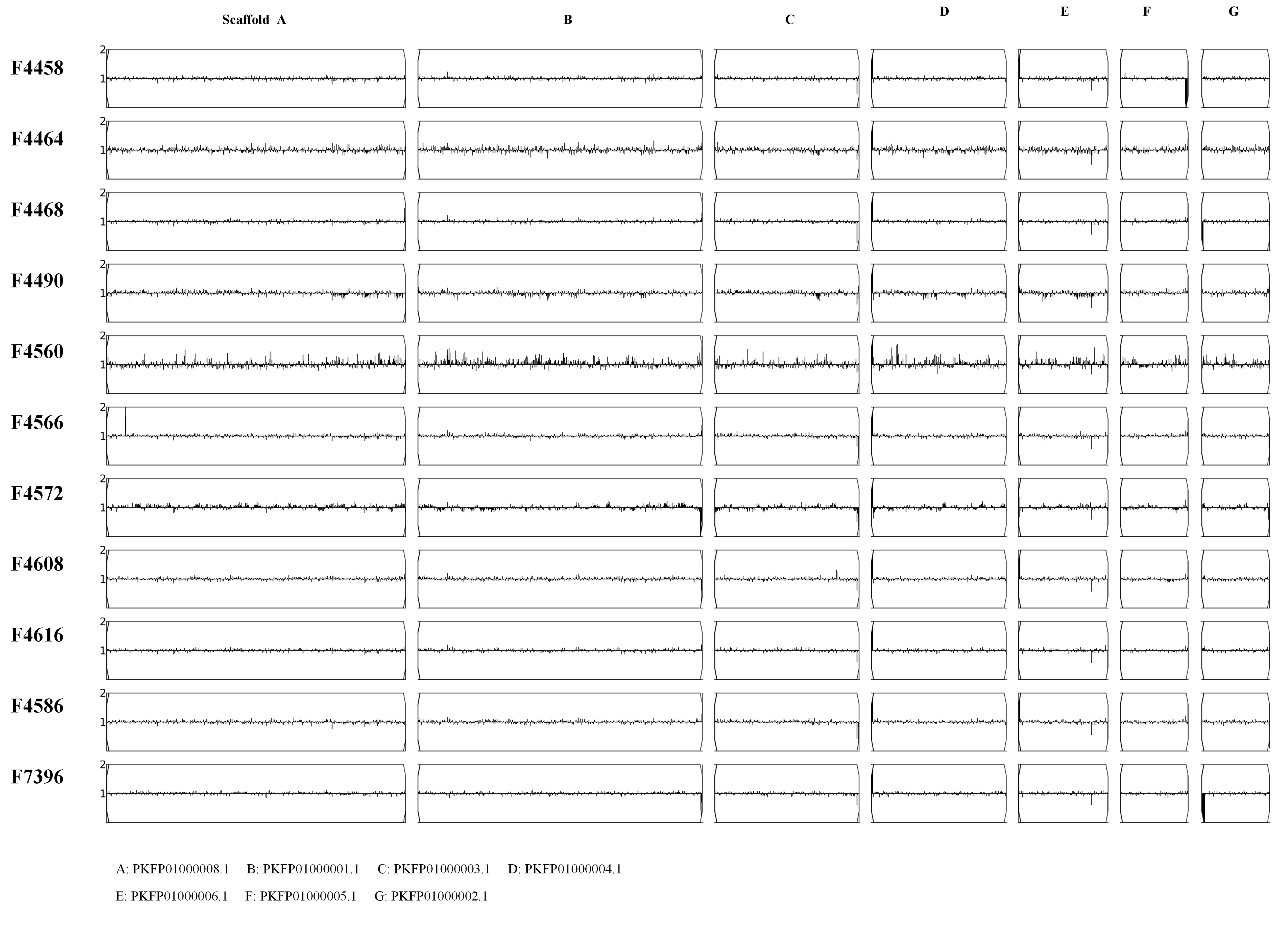

Supplement: SUPPLEMENTARY FIGURE S1 — No obvious copy number variation in Candida duobushaemulonii. [file Image_1.tif]
